# Integrated single-cell analysis reveals the regulatory network of disulfidptosis-related lncRNAs in bladder cancer: constructing a prognostic model and predicting treatment response

**DOI:** 10.3389/fonc.2025.1527036

**Published:** 2025-03-05

**Authors:** Jiafu Xiao, Wuhao Liu, Jianxin Gong, Weifeng Lai, Neng Luo, Yingfan He, Junrong Zou, Zhihua He

**Affiliations:** ^1^ The First Clinical College, Gannan Medical University, Ganzhou, Jiangxi, China; ^2^ Department of Urology, The First Affiliated Hospital of Gannan Medical University, Ganzhou, Jiangxi, China; ^3^ Institute of Urology, First Affiliated Hospital of Gannan Medical University, Key Laboratory of Urology and Andrology of Ganzhou, Ganzhou, Jiangxi, China; ^4^ Department of Urology, Zhongshan Hospital Xiamen University, School of Medicine, Xiamen University, Xiamen, China

**Keywords:** bladder cancer, disulfidptosis, lncRNA, single-cell RNA, sequencing, prognostic model, immune microenvironment

## Abstract

**Background:**

Disulfidptosis is a newly discovered form of cell death, and long non-coding RNAs (lncRNAs) play a crucial role in tumor cell growth, migration, recurrence, and drug resistance, particularly in bladder cancer (BLCA). This study aims to investigate disulfidptosis-related lncRNAs (DRLs) as potential prognostic markers for BLCA patients.

**Methods:**

Utilizing single-cell sequencing data, RNA sequencing data, and corresponding clinical information sourced from the GEO and TCGA databases, this study conducted cell annotation and intercellular communication analyses to identify differentially expressed disulfide death-related genes (DRGs). Subsequently, Pearson correlation and Cox regression analyses were employed to discern DRLs that correlate with overall survival. A prognostic model was constructed through LASSO regression analysis based on DRLs, complemented by multivariate Cox regression analysis. The performance of this model was rigorously evaluated using Kaplan-Meier analysis, receiver operating characteristic (ROC) curves, and area under the ROC curve (AUC). Furthermore, this investigation delved into the potential signaling pathways, immune status, tumor mutation burden (TMB), and responses to anticancer therapies associated with varying prognoses in patients with BLCA.

**Results:**

We identified twelve differentially expressed DRGs and elucidated their corresponding intercellular communication relationships. Notably, epithelial cells function as ligands, signaling to other cell types, with the interactions between epithelial cells and both monocytes and endothelial cells exhibiting the strongest connectivity. This study identified six DRLs in BLCA—namely, C1RL-AS1, GK-AS1, AC134349.1, AC104785.1, AC011092.3, and AC009951.6, and constructed a nomogram to improve the predictive accuracy of the model. The DRL features demonstrated significant associations with various clinical variables, diverse immune landscapes, and drug sensitivity profiles in BLCA patients. Furthermore, RT-qPCR validation confirmed the aberrant expression levels of these DRLs in BLCA tissues, affirming the potential of DRL characteristics as prognostic biomarkers.

**Conclusion:**

We established a DRLs model that serves as a predictive tool for the prognosis of BLCA patients, as well as for assessing tumor mutation burden, immune cell infiltration, and responses to immunotherapy and targeted therapies. Collectively, this study contributes valuable insights toward advancing precision medicine within the context of BLCA.

## 1 Introduction

BLCA, also known as bladder cancer, is a common urogenital tumor worldwide, with a significant increasing trend in the number of new cases each year (1, 2), llness incidence ranks ninth, while mortality rates rank thirteenth (3, 4). This growth trend is related to smoking, exposure to carcinogens, and environmental factors. Despite some countries witnessing a decrease in incidence rates through education and preventive measures, BLCA remains a global public health issue (5). The mortality rate of BLCA is closely related to tumor classification, stage of development, and the effectiveness of treatment strategies, due to its high recurrence and invasiveness (3, 6) postoperative care requires additional intervention methods (7–9). A thorough investigation of the etiology and molecular mechanisms of BLCA is crucial for developing personalized prognosis assessments and treatment strategies, which will help improve patients’ survival rates and quality of life. Therefore, further research will offer more breakthroughs for the treatment and management of BLCA, bringing hope and progress.

Disulfidptosis is a remarkable new discovery, and its definition and characteristics are fascinating. This form of cell death is triggered by specific disulfide bond stress, completely different from traditional types of cell death such as apoptosis, necrosis, and autophagy. In Disulfidptosis, the rapid depletion of intracellular NADPH and the high expression of the SLC7A11 protein under glucose deprivation are significant features, prompting profound reflections on the mechanisms that trigger Disulfidptosis (10). In cells with high expression of SLC7A11, glucose deprivation or uptake obstruction can lead to the significant uptake of cystine and its reduction to cysteine, depleting a large amount of NADPH in the process. This results in the accumulation of abnormal disulfides, ultimately triggering disulfide bond stress and rapid cell death, adding further mystery to the connection with cancer. Research indicates that SLC7A11 plays a crucial role in BLCA (11, 12), suggesting that Disulfidptosis may play an important role in BLCA, bringing therapeutic potential through this new discovery.

Research on long non-coding RNA (LncRNA) in the field of BLCA is indeed intriguing. These LncRNA play crucial roles in cellular physiology and pathological processes (13), encompassing the control of key processes such as cell proliferation, differentiation, and apoptosis (14, 15). In BLCA, specific LncRNAs such as MALAT1 (16), H19 (17), HOTAIR (18), and PTENP1 (19) have been found to be closely associated with the development and prognosis of the disease. An increasing number of studies have employed lncRNAs as biomarkers to predict therapeutic responses in bladder cancer, including models associated with ferroptosis, m6A methylation, and immune responses. These findings underscore the potential of lncRNAs not only as prognostic indicators but also as valuable tools for personalizing treatment approaches in the management of BLCA (20–22).

In this study, we examined the differential expression of disulfidptosis-related genes(DRGs) at the single-cell level. We successfully identified DRGs and subsequently conducted co-expression analyses to identify associated disulfidptosis-related long non-coding RNAs (DRLs). This investigation explores into the roles of DRLs in BLCA and establishes a model for assessing patient prognosis, overall survival (OS), tumor immune microenvironment (TIME), and sensitivity to immunotherapy and chemotherapy agents. The objective of this research is to elucidate the functions of these lncRNAs in BLCA and to provide more precise predictions and therapeutic strategies for personalized medicine.

## 2 Materials and methods

### 2.1 Data collection and compilation

Single-cell RNA sequencing (scRNA-seq) data (GSE135337) (23) were obtained from the GEO database (https://www.ncbi.nlm.nih.gov/), comprising seven BLCA tumor samples and one normal tissue sample. This dataset serves as a valuable resource for exploring the cellular heterogeneity and molecular characteristics associated with BLCA, facilitating a deeper understanding of the tumor microenvironment and the role of DRLs in this malignancy. We selected the seven BLCA tumor samples for subsequent analyses, followed by rigorous filtering and quality control procedures. We retrieved and downloaded RNA transcriptomic data, clinical information, and somatic mutation data for 431 BLCA patients from The Cancer Genome Atlas (TCGA) database (https://gdc.cancer.gov/), which included 412 tumor samples and 19 normal samples. Normal control samples were excluded from further analyses, and cases with a survival duration of less than 30 days, as well as those lacking sufficient age and tumor staging information, were also eliminated. The filtered dataset underwent normalization using log2 transformation to ensure consistency and enhance the interpretability of the subsequent analyses. Building upon previous research, we compiled a list of 27 DRGs for our analysis (10, 24).

### 2.2 Cell annotation analysis

The detailed steps for quality control of scRNA-seq data are as follows (1): We utilized the R package “Seurat” (25) for filtering and analyzing the scRNA-seq data. The filtering criteria were as follows: cells were retained if they exhibited a substantial number of detected RNA features greater than 50 and had a mitochondrial gene percentage of less than 5%. Cells meeting these criteria were included in the subsequent analyses, thereby ensuring the inclusion of high-quality data for further exploration of cellular heterogeneity and gene expression dynamics in BLCA.(2)The scRNA-seq dataset was normalized using the “NormalizeData” function in the Seurat package. (3) The “FindVariableFeatures” function was employed to identify variable features within the dataset, resulting in the selection of 3,000 variable genes.(4) Subsequently, the “ScaleData” function was applied to standardize the data, followed by principal component analysis (PCA) to identify significant principal components. The “ElbowPlot” function, utilizing the variable genes as input, was employed to determine the top 20 principal components, which were selected for subsequent Uniform Manifold Approximation and Projection (UMAP) analysis (dims = 20) (26). The “FindClusters” function was then utilized for cell clustering, allowing us to group cells based on their expression profiles.(5) The “FindAllMarkers” function was utilized to compare gene expression across different cell types, employing the Wilcoxon rank-sum test to identify differentially expressed genes between these cellular populations. Finally, the R package “singleR” (27) was integrated with manual adjustments of the cluster annotations to accurately ascertain the core cell types present in BLCA patients.

### 2.3 Cell–cell communication analysis

Cellular communication analysis is a pivotal component of single-cell sequencing data analysis, primarily aimed at uncovering the interactions, signaling pathways, and communication networks between cells. We employed the CellChat package (v 1.6.1) to infer the intercellular communication network, which included the identification of known ligands and receptors, as well as their interactions. Utilizing the CellChat package, we conducted an analysis of intercellular communication based on the expression of known ligand-receptor pairs across different cell types.

### 2.4 Selection of disulfidptosis-related lncRNAs(DRLs)

Based on the downloaded TCGA-BLCA transcriptomic expression profiles, we utilized Perl software (https://www.perl.org) to segregate the RNA transcriptomic expression data into lncRNAs and mRNAs. Subsequently, we employed the R packages ‘limma’ (28), ‘ggplot2’, ‘ggalluvial’, and ‘dplyr’ to conduct a Pearson correlation analysis (Since the gene expression data, after log2 normalization, approximates a normal distribution, and the research objective is to assess linear correlation, Pearson correlation analysis is employed (|cor|>0.4, p<0.001)), filtering for lncRNAs that co-express with DRGs. To visualize these lncRNAs, we employed a Sankey diagram, which effectively illustrates the relationships between the identified lncRNAs and DRGs. The lncRNAs highlighted in this analysis are defined as DRLs.

### 2.5 Construction of a prognostic model for DRLs

The sample size was determined based on a power analysis using the ‘PowerSurvFDR’ package in R. Assuming a hazard ratio (HR) of 1.5 for key lncRNAs, a significance level (α) of 0.05, and 80% statistical power (1-β), the minimum required sample size was calculated as 360.

A total of 394 BLCA patients with available survival data were initially randomized using the R programming language package into a training set and a testing set, with the training set consisting of 197 patients designated for model development and the testing set comprising 197 patients allocated for model validation. Subsequently, we performed chi-square tests to assess the clinical characteristics—namely, age, gender, grade, and stage of both groups to determining whether significant differences existed. A p-value of less than 0.05 was regarded as statistically significant. In the training cohort, univariate Cox regression analysis (UniCox) was employed to identify DRLs associated with OS. Following this, the ‘glmnet’ package in R was utilized to conduct least absolute shrinkage and selection operator (LASSO) analysis on the lncRNAs identified in the preceding step. This was followed by multivariate Cox regression analysis to validate the most relevant OS-associated lncRNAs and to develop a prognostic model based on key lncRNAs linked to dual sulfide death. The model calculation method is as follows: Risk score = $\sum_{i=x}^{n}\;$ Coe (genei)∗Exp(genei) (29). The risk score was calculated, and patients were divided into low-risk and high-risk groups based on the median risk score.

Coe(genei): the coefficient of each gene in the DRLs signature.
Exp (genei): the relative expression level of each gene in this study.

### 2.6 Model validation

Using R software (package “pheatmap”), we plotted the risk curves, survival status diagrams, and risk heatmaps for the training and testing groups, dividing them into high-risk and low-risk groups based on the median. With R software (packages “survival” and “survminer”), we generated the Kaplan-Meier (KM) curves for the high-risk and low-risk groups in the training set, testing set, and overall set, including OS and progression-free survival (PFS). Utilizing R software (“survival” package), we conducted univariate and multivariate independent prognostic analyses for the risk model. Employing R software (packages “survival”, “survminer”, and “timeROC” (30)), we generated the 1-year, 3-year, and 5-year ROC curves for the risk model, as well as the 5-year ROC curves for age, gender, grade, and stage, and performed comparisons. Using R software (packages “limma” and “scatterplot3d”), we performed PCA on the risk model using the limma and scatterplot3d R packages to see if it could distinguish all lncRNAs、DRLs、DRGs and all genes, and visualized the results. We used KM、ROC、and concordance index (C-index) curves or our analyses.

### 2.7 Construction of nomograms

We employed the “survival”,”regplot” and “rms” packages to generate nomograms, thereby validating the credibility of the model in predicting clinically relevant indicators for the entire cohort of BLCA patients. This serves as a useful reference tool for clinicians. Additionally, we utilized calibration curves to assess the accuracy of the predicted survival rates versus the actual survival rates of the prognostic model at 1, 3, and 5 years.

### 2.8 Clinical characteristics of the risk model

Using R software with the “survivor” and “survminer” packages, we plotted survival curves for patients stratified by gender, age, and tumor stage based on the risk model. This approach was undertaken to verify the applicability of our constructed risk model to patient subgroups with varying clinical characteristics.

### 2.9 Enrichment analysis of differentially expressed genes(DEGs)

In order to further investigate the functional and pathway enrichment of genes in different risk groups, we used the R package “limma” to obtain DEGs between the two risk groups, with |log2 fold change| > 1 and false discovery rate (FDR) < 0.05. We then utilized the clusterProfiler and other R software packages (“clusterProfiler”, “colorspace”, “stringi”, “ggplot2”, “circlize”, “RColorBrewer”, and “ggpubr”) to perform Gene Ontology (GO) (31) functional enrichment and Kyoto Encyclopedia of Genes and Genomes (KEGG) (32) pathway enrichment analysis on these DEGs. Finally, we employed gene set enrichment analysis (GSEA) with R packages to distinguish the functions and pathways between the two risk groups.

### 2.10 Immune infiltration analysis

According to the ESTIMATE algorithm (33, 34), we utilized the “ESTIMATE” package in R to assess the tumor microenvironment, and employed the “reshape2” and “ggpubr” R packages to analyze differences between the two risk groups. To ascertain the immunological characteristics of the 384 samples, we imported the expression data into CIBERSORT (35) using the “limma,” “parallel,” and “preprocessCore” packages in R to evaluate the percentage of immune cell infiltration (33). To explore the composition of immune cells in different risk groups, we compared the distribution of immune cells across the risk groups using the Wilcoxon test with the “limma,” “reshape2,” and “ggpubr” R packages.

### 2.11 Analysis of tumor mutational burden (TMB) and immune checkpoints

We calculated the TMB and mutation frequency for each sample in BLCA based on the total number of somatic base substitutions, and analyzed the differences in TMB between the high-risk and low-risk groups. The R package “Maftools” (36) was utilized to create the waterfall plot of mutations in DEGs. Patients with BLCA were divided into two groups according to the median TMB score, and survival analysis was conducted to explore the impact of TMB on patients’ OS.

### 2.12 Immunotherapy and drug sensitivity prediction

We utilized the Tumor Immune Dysfunction and Exclusion (TIDE) (37) platform(http://tide.dfci.harvard.edu/)to predict the immunotherapy outcomes for patients with BLCA (37, 38). By comparing TIDE scores, we analyzed the differences in immune therapy responses between high-risk and low-risk groups. The R packages limma, oncoPredict, and parallel were employed to assess the half-maximal inhibitory concentration (IC50) values of chemotherapy drugs. The requisite training set was sourced from the Genomics of Drug Sensitivity in Cancer (GDSC) database and downloaded from the oncoPredict Open Science Framework repository(https://osf.io/c6tfx/) (39). We utilized the calcPhenotype function to obtain drug sensitivity scores for each patient. The differences in responses to multiple drugs between the high-risk and low-risk groups were compared based on the drug sensitivity scores (40).

### 2.13 Validation of lncRNAs in the model

LncRNA data obtained from the TCGA-BLCA database were subjected to differential analysis to evaluate the expression of relevant lncRNAs. Additionally, tumor and paired adjacent tissue samples were collected from ten patients who underwent radical cystectomy for BLCA at the First Affiliated Hospital of Gannan Medical University. All patients were pathologically confirmed to have BLCA. All samples in this study were obtained with the informed consent of each patient and were authorized by the hospital’s ethics committee, in accordance with the Declaration of Helsinki. The expression of the relevant lncRNAs was detected using real-time fluorescent quantitative PCR (qRT-PCR).

### 2.14 qRT-PCR

Following the manufacturer’s experimental protocol, total RNA was extracted from the collected tissue samples using an RNA extraction reagent. The total RNA was then reverse transcribed into complementary DNA (cDNA) according to the reverse transcription steps. Nuclease-Free Water, primers, cDNA, and Universal Blue SYBR Green qPCR Master Mix were mixed in proportion and real-time quantitative PCR (qRT-PCR) was performed using a PCR detection system with the following program and parameters: initial denaturation (95°C, 30 seconds), denaturation (95°C, 15 seconds), annealing/extension (60°C, 30 seconds). A total of 40 cycles were conducted. Data were normalized using GAPDH as the control group. The relative quantification method (2−ΔΔCt) was used to assess the relative expression of the lncRNA. Primer Sequences are presented in **Table 1**.

**Table 1:** Sequence of primers for qPCR.

Primer name	Direction	Primer sequence 5’—3’
AC134349.1	Forward	TCAAGACCCTCCACTGATACAAGA
AC134349.1	Reverse	TAGCAACAGATGGCTTTCACCC
AC011092.3	Forward	AGATGCTATGCAGCCTAACTTTACA
AC011092.3	Reverse	GAACTGCCTGGTTATGGAATGG
AC009951.6	Forward	GGATTGGATTGCGAGTCTGC
AC009951.6	Reverse	GCAAGCAAAGGGTGATAAAGG
C1RL-AS1	Forward	CGTCTGTGGTGAGAAGCCTGAT
C1RL-AS1	Reverse	GCTTTCTGTTCCACTGTGCTCTT
GK-AS1	Forward	ATTCCCTCCCTTCCTGACTTTA
GK-AS1	Reverse	GCTTCCAGGTTCATTCAGGTTAT
AC104785.1	Forward	GTGTTCTTAGGCTCCTCTTGGC
AC104785.1	Reverse	TGTTAGTGGGGGCAAGAAATG
GAPDH	Forward	GGAAGCTTGTCATCAATGGAAATC
GAPDH	Reverse	TGATGACCCTTTTGGCTCCC

### 2.15 Statistical analysis

The t-test was used to compare continuous variables between two groups, while categorical variables were analyzed using the chi-square test. Univariate and multivariate survival analyses were performed using Cox regression analysis. The log-rank test was employed for analyzing OS data. All data were analyzed using R version 4.4.0 or GraphPad Prism 9.

This study screened DRGs through single-cell sequencing, constructed a DRLs model using TCGA data, and evaluated its prognostic value through KM, ROC curves, and C-index. Ultimately, the study verified the association of the model with the immune microenvironment and treatment response.

## 3 Results

**Figure 1:**
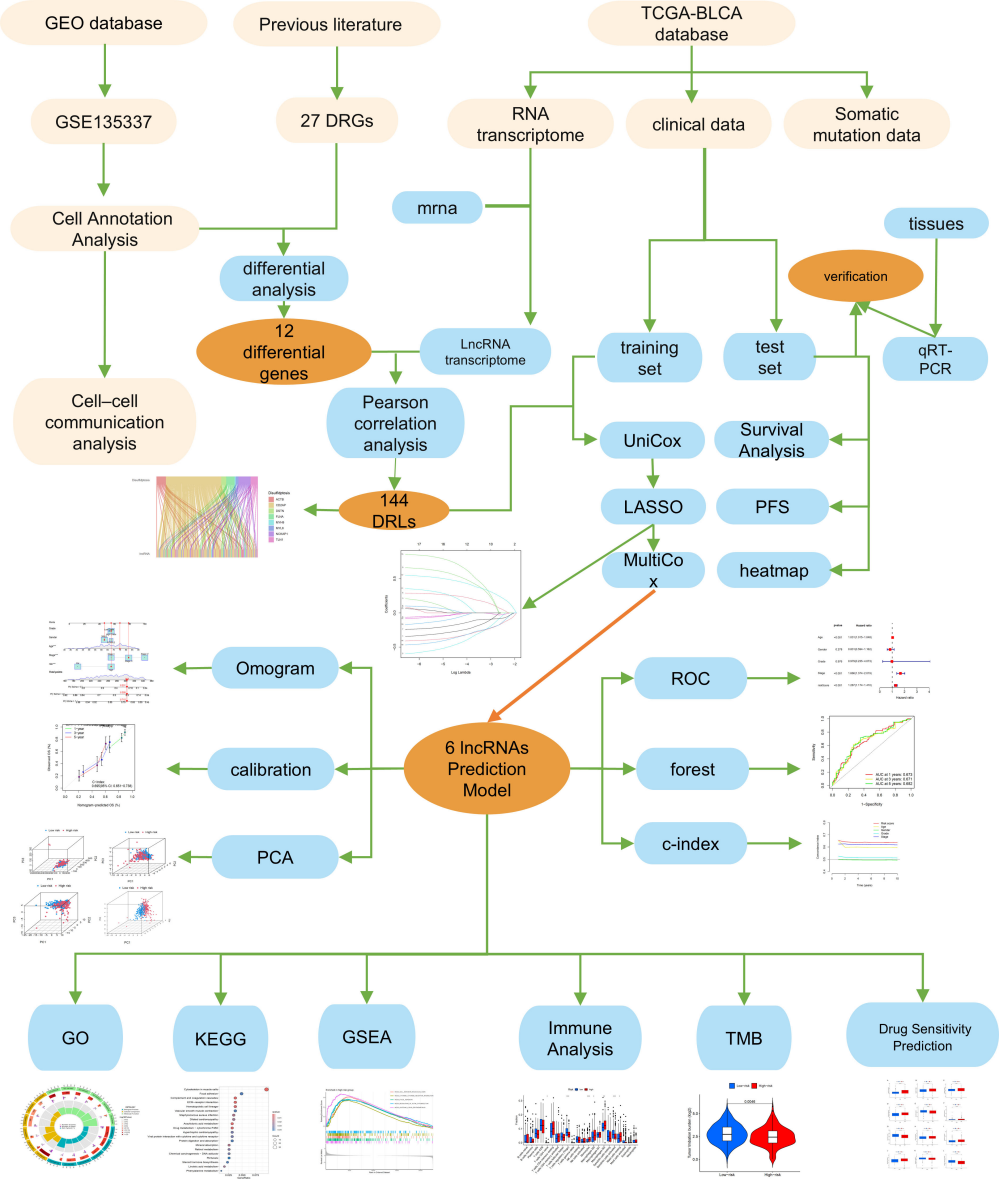
The flow chart of the study. *, p < 0.05; **, p < 0.01; ***, p < 0.001.

The Technical Roadmap of This Study (**Figure 1**).

### 3.1 Cell annotation and cell trajectory analysis

**Figure 2:**
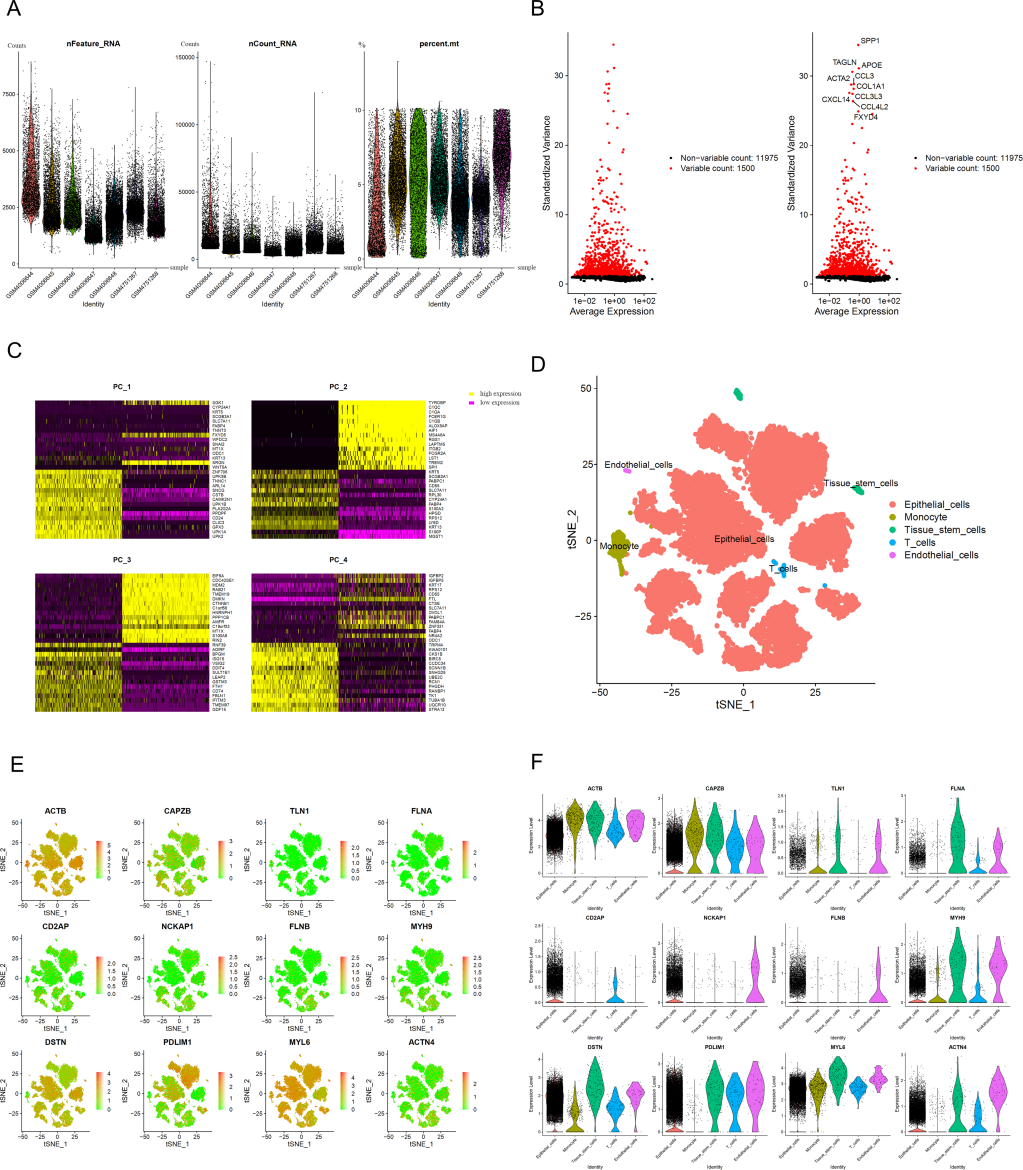
Single-cell analysis results **(A)**, Gene count、sequencing depth and percentage of mitochondrial genes. **(B)** The 1500 most variable genes and the 10 most variable genes. **(C)** Heatmap of PCA (Principal Component Analysis). **(D)** Cluster map after cell annotation. **(E)** Scatter plots for 12 DRGs (Cell types are shown in **D**). **(F)** Violin plots for 12 DRGs.

We performed quality control on the scRNA-seq data, discarding low-quality cells and genes, as shown in **Figure 2A**: the number of genes per cell was predominantly below 5000, the number of transcripts was below 4000, the percentage of mitochondrial content was low in each sample, and the top 10 highly variable genes were marked (**Figure 2B**). Principal Component Analysis was performed, and the heatmap of the related expressed genes was obtained (**Figure 2C**). After dimensionality reduction with UMAP, 20 clusters were identified, and the “singleR” package was used to annotate the clusters, resulting in 5 core cell types, including endothelial cells, tissue stem cells, epithelial cells, monocytes, T cells, etc. A detailed cell count for each cell type is displayed in **Figure 2D**, showing that endothelial cells have the highest proportion, with other cells having lower proportions. The FindAllMarkers algorithm was then employed to retrieve differentially expressed genes for each core cell type (with filtering criteria of an absolute logFC greater than 1 and adjPvalFilter < 0.05), obtaining a comprehensive list of differential genes. Ultimately, the differential expression of 12 genes was obtained (**Figures 2E, F**). The differential genes were intersected with DRGs, and the intersected genes were visualized and subjected to subsequent prognostic correlation analysis.

### 3.2 Cell-to-cell communication analysis

**Figure 3:**
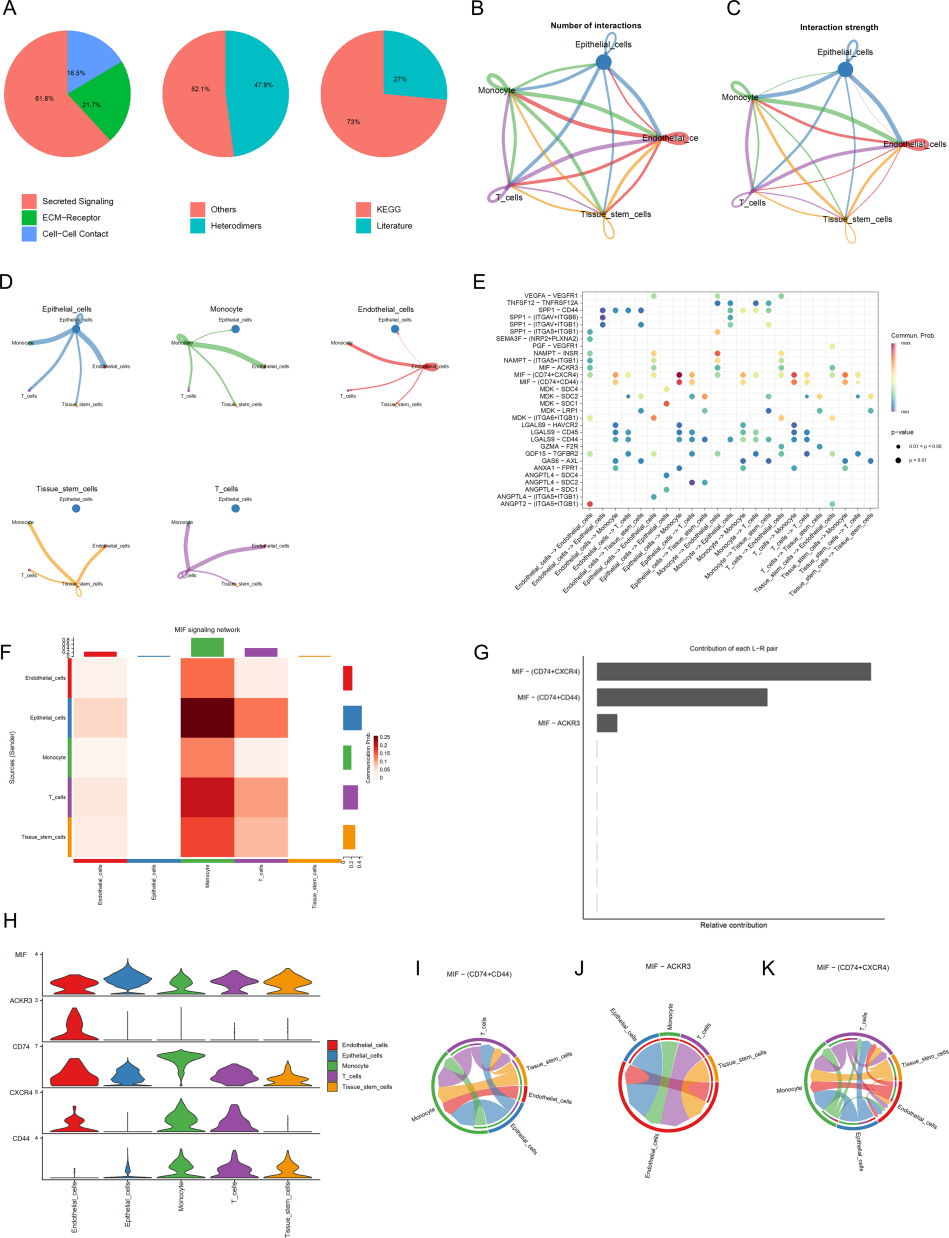
Cell Communication Analysis **(A)** Percentage graph of receptor-ligand pair types. **(B)** Relationship graph of interaction quantity. **(C)** Relationship graph of interaction strength. **(D)** Communication network graph for individual cell types. **(E)** Bubble chart of receptor-ligand pairs FHeatmap of cell communication. **(F)** Represents the heatmap of cell communication **(G)** Analysis graph of receptor-ligand pairs. **(H)** Expression levels of interaction genes. **(I-K)** Cellular communication maps at the receptor-ligand pair level.

In order to construct a cell communication map for BLCA, we created an object using CellChat and utilized the CellChatDB.human database to examine the types of ligand and receptor pairs (**Figure 3A**). We then calculated the probabilities of cell communication, filtering out cell communications involving fewer than 10 cells to obtain the cell-cell communication relationships (**Figures 3B, C**). As shown in **Figure 3D**, the results of single-cell communication indicate that epithelial cells can act as ligands to send signals to other cells, with the strongest interactions observed with Monocytes and Endothelial Cells. A bubble chart was constructed (**Figure 3E**) to detail the intercellular communication between the five cell types, thereby gaining a deeper understanding of their interactions. For instance, in Epithelial_cells−>Monocyte, Tissue_stem_cells−>Monocyte, T_cells−>Monocyte, tissue stem cells−>Monocyte, Endothelial_cells−>Monocyte, and Epithelial_cells−> T_cells, various signal pairs are involved, such as MIF-(CD74+CD44) and MIF- (CD74+CXCR4), Macrophage migration inhibitory factor (MIF) promotes the proliferation and survival of tumor cells by binding to CD74 and CD44. In breast cancer, high expression of MIF is associated with the invasiveness of tumor cells and poor prognosis. MIF activates the PI3K/AKT signaling pathway, inhibits apoptosis in tumor cells, and facilitates tumor growth and metastasis (41). MIF facilitates the invasion and metastasis of tumor cells by binding to CXCR4. In colorectal cancer, MIF promotes the migration and invasion of tumor cells by activating the CXCR4 signaling pathway, thereby increasing the risk of tumor metastasis (42).We further identified the pathways involving core genes and the interaction relationships between the cells. In this step, we mainly determined three pairs of receptor-ligand pairs, as shown in **Figures 3F, G**. In **Figure 3H**, the expression levels of interaction genes in various cell types are clearly visible. Finally, we also obtained a horizontal communication map for the receptor-ligand pairs (**Figures 3I–K**). For example, MIF-(CD74+CXCR4) is the most complex in cell communication and may become a therapeutic target.

### 3.3 Screening of DRLs

**Figure 4:**
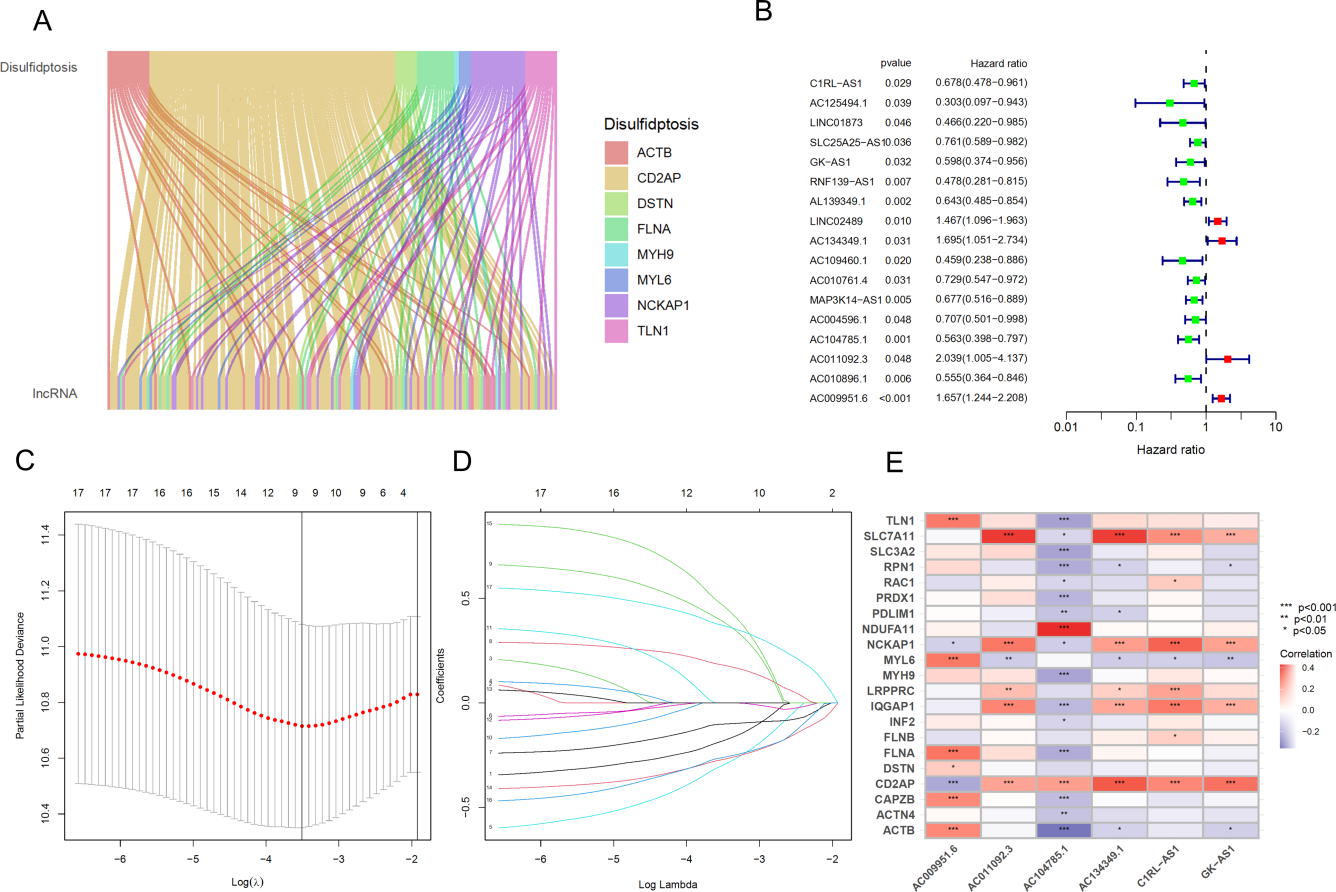
Identification and prognostic model construction of DRLs in bladder cancer. **(A)** Sankey diagram showing the correlation between DRGs and the expression of 144 lncRNAs. **(B)** Univariate Cox regression analysis to evaluate 17 prognostic-related lncRNAs. **(C)** Lasso regression curve for 9 lncRNAs. **(D)** Ten-fold cross-validation of variables in the LASSO model. **(E)** Expression correlation between the 6 lncRNAs used for model construction and DRGs. *p < 0.05; **p < 0.01; ***p < 0.001.

RNA-seq data from BLCA patients were downloaded from the TCGA database, normalized, and mRNA and lncRNA expression profile data were extracted separately according to gene type. After intersecting the differential genes in cells with disulfidptosis genes, 12 disulfidptosis genes were obtained, and a Pearson correlation analysis was performed with lncRNAs to identify 144 lncRNAs (**Figure 4A**), and a Sankey diagram was constructed. Additionally, as shown in **Figure 4B**, 17 prognostic-related lncRNAs were identified using UniCox analysis in the training set. Nine lncRNAs were identified using LASSO logistic regression analysis (**Figures 4C, D**), and finally, a multifactorial regression analysis yielded 6 lncRNAs for model construction, with the formula as follows: Riskscore = ExpC1RL-AS1*(-0.460404324) + ExpGK-AS1*(-0.560636669)+ ExpAC134349.1*(0.614635779) + ExpAC104785.1*(-0.472889887)+ ExpAC011092.3*(0.743568001) + ExpAC009951.6*(0.482146255).

Furthermore, we generated a heatmap of these 6 hub lncRNAs and DRGs (**Figure 4E**).

### 3.4 Validation of disease prediction model

**Figure 5:**
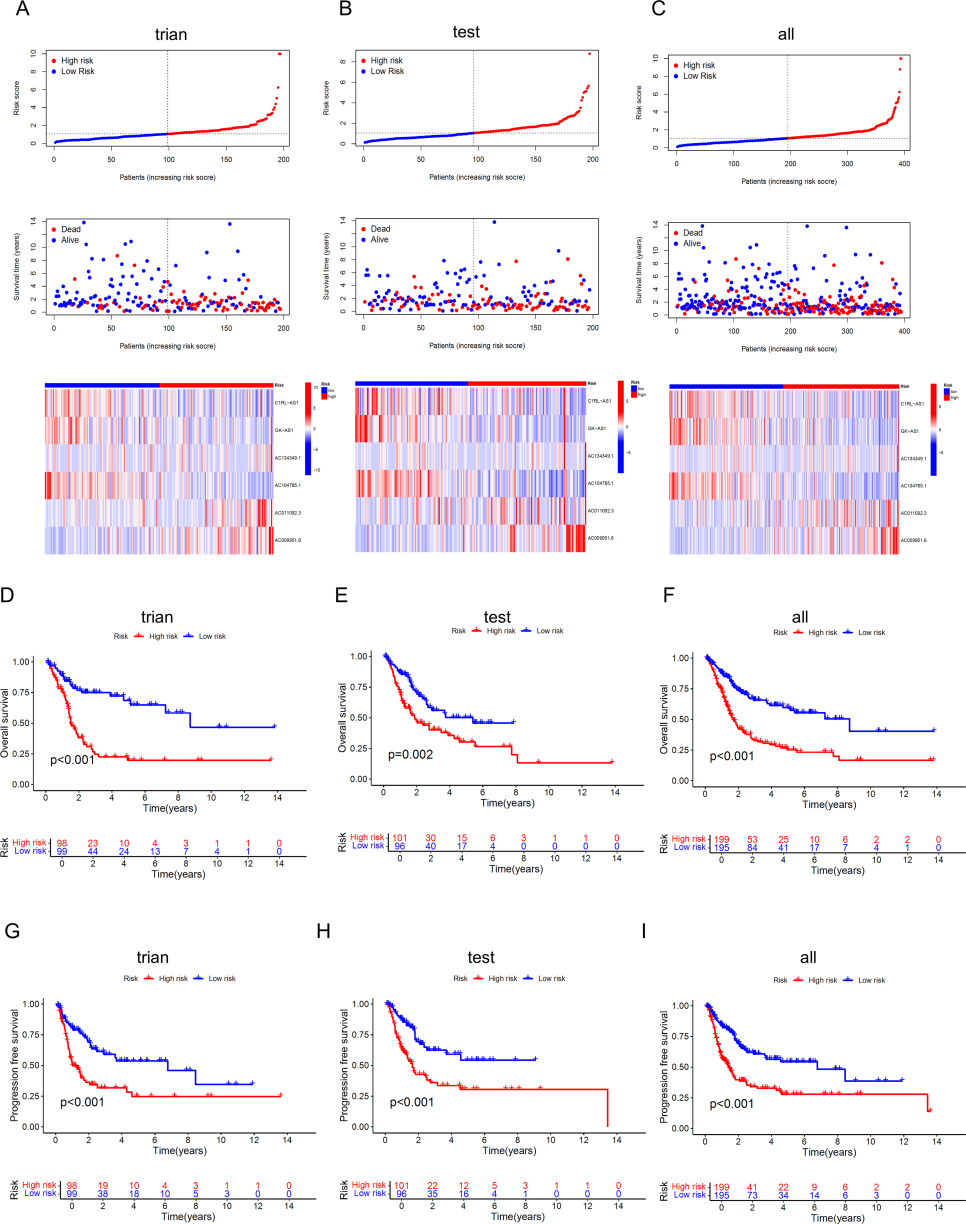
Identification and evaluation of the prognostic value of the DRLs model. **(A-C)** Curve charts of risk scores from low to high for each BLCA patient in the training set, validation set, and overall set, survival status of BLCA patients sorted from low to high, and heatmap of the correlation between the 6 key lncRNAs and risk scores. **(D-F)** KM curves for OS of high-risk and low-risk patients in the training set, validation set, and overall set, indicating that the model has significant survival discrimination capabilities. **(G-I)** KM curves for progression-free survival of high-risk and low-risk patients in the training set, validation set, and overall set.

We randomly divided 394 BLCA patients with survival information into a training set and a testing set in a 1:1 ratio. The t-test results for the clinical data (age, gender, grade, stage, T, M, N) of the two groups are shown in **Table 2**. The training set was used to build the model, and the testing set was used to validate the model. Based on the patient risk scores obtained from the previous steps, patients were divided into high-risk and low-risk groups using the median risk score, and risk curves, survival status curves, and risk heatmaps were plotted for the training set, testing set, and overall set (**Figures 5A–C**). As the risk score increased, the number of patient deaths also increased, which is consistent with the predictions of our model. The risk heatmap shows that AC134349.1, AC011092.3, and AC009951.6 in the training set, testing set, and overall set are positively correlated with the risk score, while C1RL-AS1, GK-AS1, and AC104785.1 show an opposite relationship, indicating that the predictive performance of this risk model is consistent. We conducted KM curve analysis for OS (**Figures 5D–F**) and PFS (**Figures 5G–I**), and it can be seen that the model has significant survival discrimination capabilities.

**Table 2:** Results of t-test for clinical data of train and test sets.

Covariates	Type	Total	Test	Train	Pvalue
**Age**	≤65	158 (40.1%)	80 (40.61%)	78 (39.59%)	**0.9181**
	>65	236 (59.9%)	117 (59.39%)	119 (60.41%)	
**Gender**	Female	103 (26.14%)	55 (27.92%)	48 (24.37%)	**0.4915**
	Male	291 (73.86%)	142 (72.08%)	149 (75.63%)	
**Grade**	High Grade	373 (94.67%)	185 (93.91%)	188 (95.43%)	**0.8007**
	Low Grade	18 (4.57%)	10 (5.08%)	8 (4.06%)	
	unknow	3 (0.76%)	2 (1.02%)	1 (0.51%)	
**Stage**	Stage I	2 (0.51%)	0 (0%)	2 (1.02%)	**0.5691**
	Stage II	123 (31.22%)	62 (31.47%)	61 (30.96%)	
	Stage III	138 (35.03%)	69 (35.03%)	69 (35.03%)	
	Stage IV	129 (32.74%)	65 (32.99%)	64 (32.49%)	
	Unknown	2 (0.51%)	1 (0.51%)	1 (0.51%)	
**T**	T0	1 (0.25%)	1 (0.51%)	0 (0%)	**0.7428**
	T1	3 (0.76%)	1 (0.51%)	2 (1.02%)	
	T2	112 (28.43%)	52 (26.4%)	60 (30.46%)	
	T3	190 (48.22%)	97 (49.24%)	93 (47.21%)	
	T4	56 (14.21%)	27 (13.71%)	29 (14.72%)	
	unknow	32 (8.12%)	19 (9.64%)	13 (6.6%)	
**N**	N0	228 (57.87%)	114 (57.87%)	114 (57.87%)	**0.7656**
	N1	44 (11.17%)	24 (12.18%)	20 (10.15%)	
	N2	75 (19.04%)	36 (18.27%)	39 (19.8%)	
	N3	6 (1.52%)	2 (1.02%)	4 (2.03%)	
	Unknown	41 (10.41%)	21 (10.66%)	20 (10.15%)	
**M**	M0	188 (47.72%)	100 (50.76%)	88 (44.67%)	**1**
	M1	10 (2.54%)	5 (2.54%)	5 (2.54%)	
	Unknown	196 (49.75%)	92 (46.7%)	104 (52.79%)	

Bold values indicate statistical significance at the p < 0.05 level.

**Figure 6:**
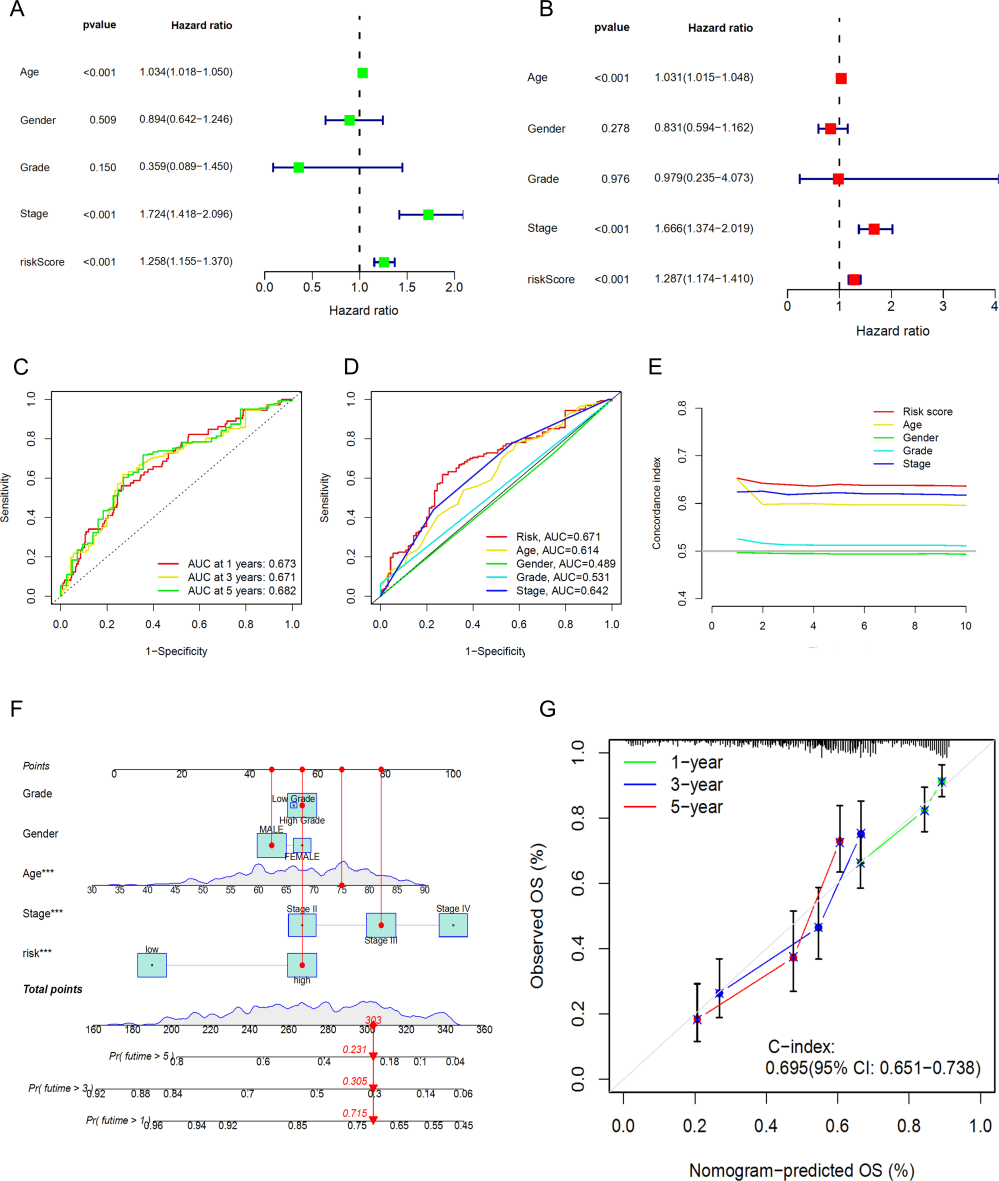
Prognostic value of the DRLs model and construction and validation of the nomogram. **(A)** Univariate Regression Analysis. **(B)**,Multivariate Regression Analysis. **(C)** ROC Curve Analysis for 1-year, 3-year, and 5-year Survival. **(D)** ROC Curve Analysis for Clinical Characteristics. **(E)** Concordance Index Analysis for Clinical Characteristics. **(F)** Nomogram. **(G)** Calibration Curves for 1-year, 3-year, and 5-year Survival.

To verify whether our prognostic model has independence compared to clinical characteristics, we further included four clinical characteristics of BLCA patients, Age, Gender, Grade, and Stage, and conducted an independent prognostic analysis. The results of univariate and multivariate regression analyses showed that Age, Stage, and riskscore are independent prognostic indicators for BLCA patients (**Figures 6A, B**), with the P-values for riskscore all being less than 0.001, the univariate hazard ratio at 1.258 (1.155-1.370), and the multivariate hazard ratio at 1.287 (1.174-1.410). However, the anomaly in the hazard ratio might be related to collinearity or a small sample size within subgroups, and thus requires validation with a larger sample size. Subsequently, we performed ROC curve analysis to calculate the AUC (Area Under the Curve) values of the prognostic model. The AUC values for the 1-year, 3-year, and 5-year survival rates were 0.673, 0.671, and 0.682, respectively (**Figure 6C**), indicating that the risk model has good predictive performance. Additionally, the AUC for riskScore was 0.671, which is significantly higher than other clinical variables in predicting the prognosis of BLCA patients, such as Age (AUC=0.614), Gender (AUC=0.489), Grade (AUC=0.531), and Stage (AUC=0.642) (**Figure 6D**). The C-index (concordance index) also showed the same predictive performance (**Figure 6E**). We then constructed a nomogram based on the risk model and clinical data that includes clinical pathological variables and features (**Figure 6F**) to further determine the prognosis of BLCA patients (Stage I types were excluded due to the small number of cases). The nomogram predicted the 1-year, 3-year, and 5-year overall survival (OS) and generated corresponding calibration curves (**Figure 6G**), with good consistency between the predicted and observed values for the predicted probability of OS.

**Figure 7:**
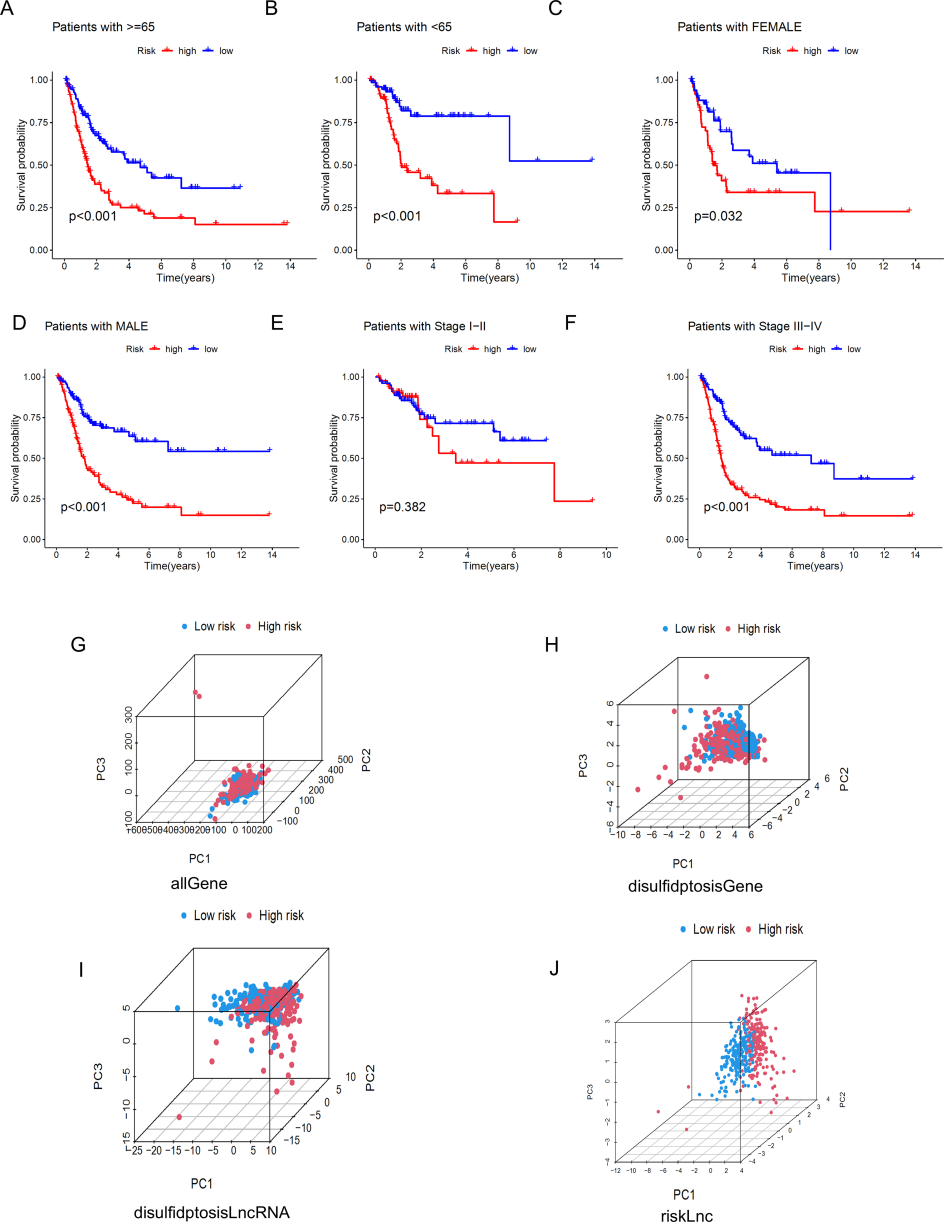
Kaplan-Meier survival curves and PCA analysis demonstrate the prognostic value of the risk model in BLCA patients, stratified by various clinical characteristics. **(A-F)** These figures show the KM curves for low-risk and high-risk BLCA patients, categorized based on different clinical characteristics. **(G-J)** Represent PCA analyses for all genes, disulfidptosis genes, disulfidptosis lncRNAs, and risk lncRNAs, respectively.

To further understand whether the risk score also has the ability to distinguish high-risk and low-risk groups in different clinical characteristic subgroups, we divided Age into Age≥65 years and Age<65 years, Gender into FEMALE and MALE, and Stage into Stage I-II and Stage III-IV groups, and performed KM curve analysis for each. The results shown in **Figures 7A–F** indicate that, except for the Stage I-II group, the OS of patients in the high-risk group was lower than that of patients in the low-risk group. These results suggest that the low-risk group has a more significant survival advantage. Overall, our analysis proves that the prognostic model is a reliable clinical prediction tool. By performing PCA on BLCA patients, we can observe whether the lncRNAs constructed in the model can clearly distinguish patients in the high-risk and low-risk groups. The three-dimensional visualization of the PCA results (**Figures 7G–J**) shows that, in **Figure 7J**, most patients in the high-risk group are located in the upper right of the three-dimensional graph, while patients in the low-risk group are in the upper left area. Therefore, the lncRNAs involved in the model construction can effectively distinguish patients in the high-risk and low-risk groups.

### 3.5 Enrichment analysis of DEGs

**Figure 8:**
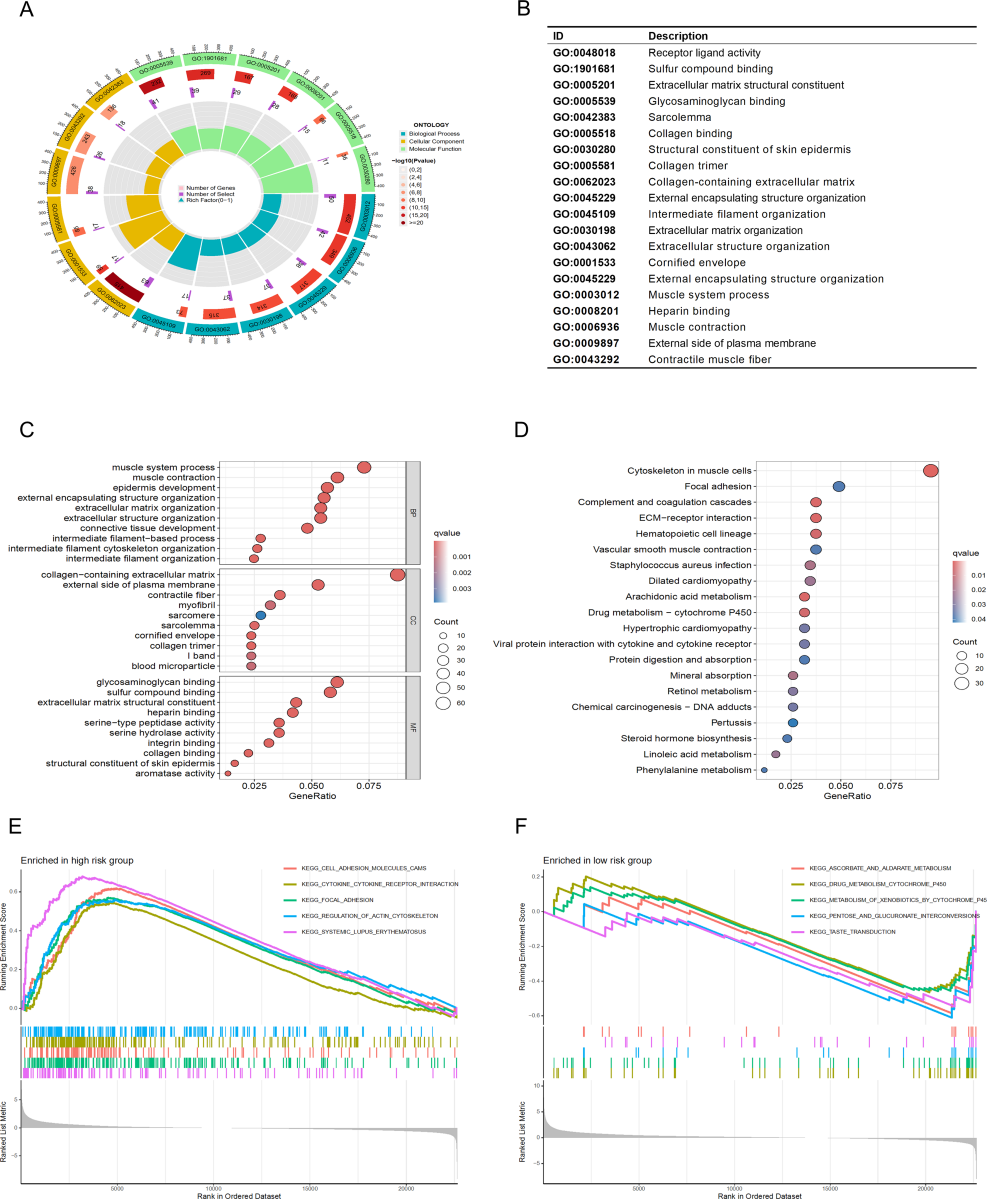
Functional analysis of the risk model.(“BP:(Biological Process);CC:(Cellular Component);MF:(Molecular Function). The significance of enrichment has been adjusted using the Benjamini-Hochberg method, with a false discovery rate (FDR) less than 0.05; the intensity of the color represents the -log10(FDR) value.) **(A, B)** GO analysis demonstrates enrichment in molecular biological processes (BP), cellular components (CC), and molecular functions (MF). **(C, D)** KEGG pathway analysis shows significantly enriched pathways. **(E, F)** GSEA analysis based on the KEGG pathway database for the high-risk and low-risk groups.

To further understand the functional correlations and signaling pathways between the high-risk and low-risk groups, we conducted GO functional enrichment analysis and KEGG pathway enrichment analysis on the DEGs between the high-risk and low-risk groups. The GO enrichment circular diagram shows that the outermost circle represents the GO enrichment ID, the second circle represents the number of genes associated with each GO term, the third circle represents the number of enriched differentially expressed genes in each GO term, and the fourth circle represents the proportion of enriched genes. The redder the color, the more significant the enrichment of differentially expressed genes. It can be seen that the proportion of genes enriched in GO:0003012, GO:0062023, and GO:0009897 is relatively high (**Figures 8A, B**). The results of the GO functional enrichment analysis (**Figure 8C**) indicate that in terms of molecular function (MF), the main focus is on intermediate filament organization, intermediate filament cytoskeleton organization, intermediate filament−based process, and connective tissue development. In cellular component (CC), the main focus is on blood microparticle, I band, collagen trimer, cornified envelope, sarcolemma, sarcomere, myofibril, contractile fiber, external side of plasma membrane, and collagen−containing extracellular matrix. In biological process (BP), the main focus is on integrin binding, serine hydrolase activity, serine−type peptidase activity, heparin binding, extracellular matrix structural constituent, sulfur compound binding, and glycosaminoglycan binding. The KEGG pathway analysis results show that the focus is mainly on the Cytoskeleton in muscle cells, Focal adhesion, Complement and coagulation cascades, and ECM-receptor interaction pathways, among which the cytoskeleton, adhesion, migration, and proliferation are important mechanisms for cell movement functions and may be related to cancer cell proliferation and migration (**Figure 8D**). GSEA analysis was also performed (**Figures 8E, F**), cell adhesion molecules cams, focal adhesion, regulation of actin cytoskeleton, and systemic lupus erythematosus are the top 5 significantly enriched items in the high-risk group, while in the low-risk group, ascorbate and aldarate metabolism, drug metabolism cytochrome p450, metabolism of xenobiotics by cytochrome p450, pentose and glucuronate interconversions, taste transduction are the top 5 significantly enriched cellular processes.

### 3.6 Immune infiltration, tumor immune microenvironment tumor (TME) and mutational landscape

**Figure 9:**
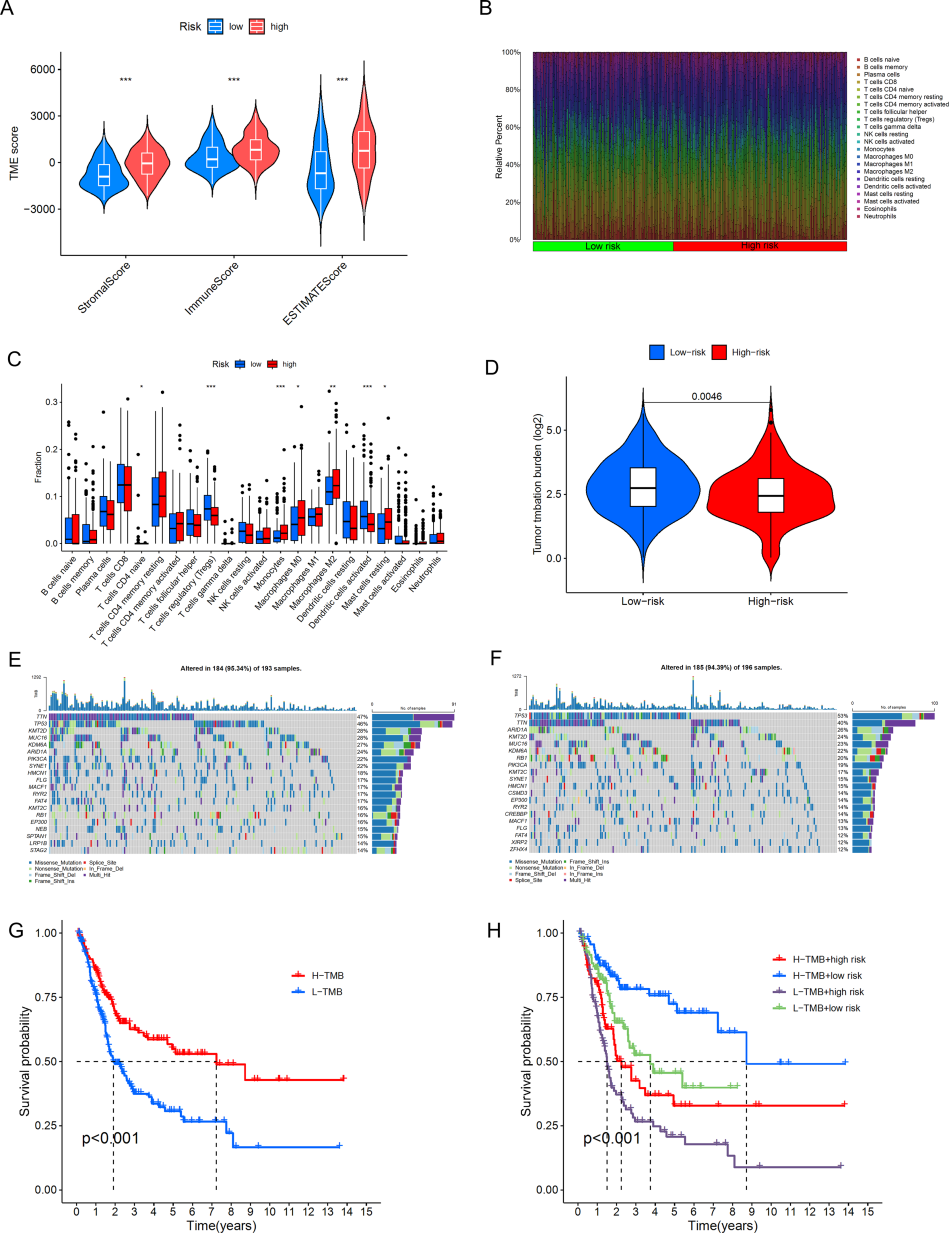
Differences in the tumor immune microenvironment between the low-risk and high-risk groups. **(A)** Violin plots comparing StromalScore, ImmuneScore, and ESTIMATEScore between the low-risk and high-risk groups. **(B)**, Proportions of 22 tumor-infiltrating immune cell types in BLCA patients. **(C)** Differences in various types of immune cells between the high-risk and low-risk groups. **(D)** Abundance ratio of immune cells in BLCA samples. **(E, F)** KM analysis of OS for patients classified by TMB status and risk score. **(G, H)** KM analysis of OS for patients categorized by combining TMB status and risk score *, p < 0.05; **, p < 0.01; ***, p < 0.001.

The TME plays a crucial role in determining tumor progression (43). We further analyzed this using the CIBERSORT algorithm. From **Figure 9A**, we can observe the stromal cell scores and the integration of stromal and immune cell scores in the tumor microenvironment for both high-risk and low-risk groups, such as StromalScore (stromal cells in tumor tissue), ImmuneScore (immune cell infiltration in tumor tissue), and ESTIMATEScore (the sum of stromal and immune scores). The results show that the high-risk group has higher stromal cell scores and stromal-immune cell integration scores, indicating a higher abundance of stromal cells in this group. Additionally, we displayed the infiltration levels of immune cell populations between high-risk and low-risk groups using a bar chart (**Figure 9B**). The high-risk group contains higher amounts of Monocytes, Macrophages M0, Macrophages M2, and Mast cells resting, while the low-risk group shows a positive correlation with T cells regulatory (Tregs) and Dendritic cells activated (**Figure 9C**). Monocytes and Macrophages M0/M2 are key cells in inflammatory responses, and their accumulation in tissues may be associated with inflammatory diseases or an immunosuppressive state in the tumor microenvironment. M2-type macrophages are particularly associated with an anti-inflammatory and tumor growth-promoting environment. The high content of Tregs in the low-risk group may be related to a stronger immunosuppressive environment; Tregs help maintain immune tolerance and prevent excessive immune responses, but in the tumor microenvironment, they may also aid the tumor in evading immune surveillance. These results indicate that in our model, as the risk score increases, the tumor immune microenvironment may be disrupted, leading to tumor progression and poorer overall survival. Based on these findings, we infer that reduced immune cell infiltration and activity lead to poor prognosis in BLCA patients, who can be distinguished by our DRLs model.

We further conducted a correlation analysis between the riskscore and gene mutations. TMB (Tumor Mutational Burden) refers to the number of mutations in tumor cells per million base pairs (megabase, Mb). The level of TMB has significant clinical implications in oncology, and subsequently, we performed an analysis of the distribution of somatic mutations in BLCA patients in the two risk groups. The results shown in **Figure 9D** indicate that, in terms of overall tumor mutational burden, we observed a higher mutation frequency in the low-risk group. In the high-risk group (**Figure 9E**), TTN, TP53, KMT2D, MUC16, KDM6A, ARID1A, PIK3CA, SYNE1, HMCN1, and FLG are the top 10 most frequently mutated genes. In the low-risk group (**Figure 9F**), TP53, TTN, ARID1A, KMT2D, MUC16, RB1, PIK3CA, KMT2C, SYNE, and HMCN1 are the top 10 most frequently mutated genes. A comprehensive analysis (**Figures 9G, H**) shows that the survival rate in the high TMB group is significantly higher than that in the low TMB group, and the difference is statistically significant (p<0.001). There is also a clear difference in survival rates between the low-risk and high-risk groups, and the difference is significant (p<0.001). This also suggests that high TMB may be associated with immune cell infiltration in the tumor microenvironment; these immune cells can recognize tumor cells and participate in tumor clearance (44).

### 3.7 Drug sensitivity analysis

**Figure 10:**
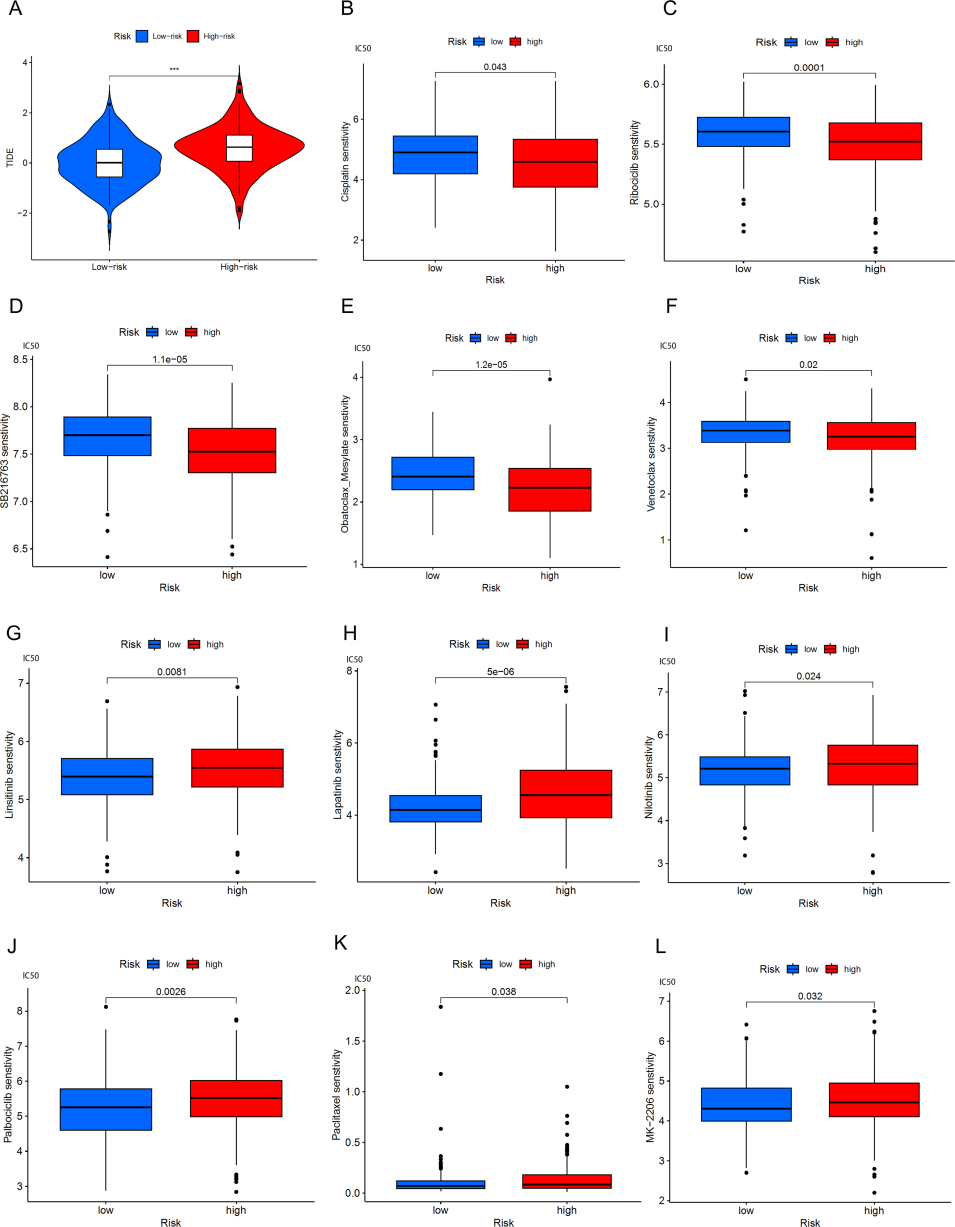
BLCA patients’ response to immune checkpoint blockade and other antitumor treatments. **(A)** Violin plots show the difference in TIDE scores between high-risk and low-risk BLCA groups. **(B-F)** Drugs with better efficacy in the high-risk group. **(G-L)** Drugs with better efficacy in the low-risk group, ***p < 0.001.

We found that the TIDE score was significantly increased in high-risk BLCA patients (**Figure 10A**), which may indicate that these patients have a higher immune evasion capability. To assess the association between the risk model and drug sensitivity, we used the oncoPredict package to evaluate the IC50 values of various drugs in high and low-risk groups of BLCA patients, as shown in the results (**Figures 10B–L**). High-risk BLCA patients showed better efficacy against Cisplatin, Ribociclib, SB216763, and Obatoclax Mesylate, Venetoclax, and higher resistance to Cytarabine, Lapatinib, Linsitinib, Nilotinib, MK-2206 (AKT inhibitor), Rapamycin (mTOR inhibitor), and Navitoclax. These results suggest that our model can provide personalized drug sensitivity predictions for BLCA patients, which can help guide clinicians in choosing the most appropriate drugs and treatment plans to improve therapeutic outcomes and reduce the development of drug resistance.

### 3.8 Validation of lncRNA expression

**Figure 11:**
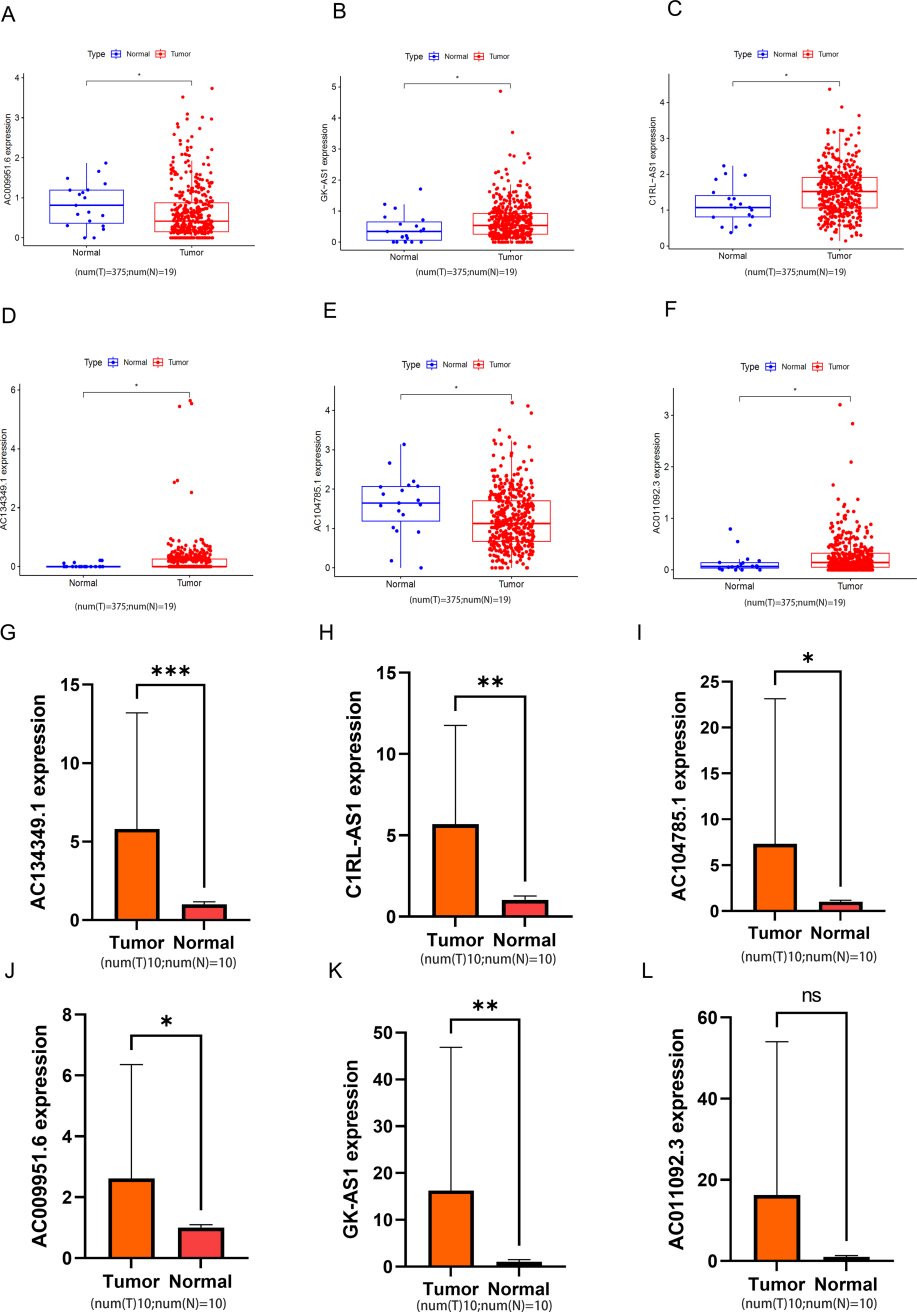
The relative expression levels of the 6 lncRNAs. **(A-F)** In the TCGA database, the expression levels of 6 types of lncRNA in bladder tumor tissue and normal bladder tissue. **(G-L)** Relative normalized expression of six DRLs in bladder tumor tissues compared to adjacent normal tissues. *p < 0.05; **p < 0.01; ***p < 0.001; ns, no significance.

To validate the reliability of the six lncRNAs constituting the risk model, we analyzed their corresponding expression levels using data downloaded from the TCGA database. The results of the six lncRNAs identified in cancerous and non-cancerous tissues of BLCA patients from the TCGA database are shown in **Figures 11A–F**. Compared to normal samples, the expression levels of AC104785.1 and AC009951.6 were higher in BLCA. Conversely, GK-AS1, C1RL-AS1, AC134349.1, and AC011092.3 exhibited higher expression levels than adjacent non-cancerous tissues. We further investigated the relative expression levels of the six lncRNAs using qRT-PCR on 10 pairs of BLCA tumor tissues and corresponding adjacent normal tissues. As shown in **Figures 11G–L**, all six lncRNAs exhibited higher expression in tumor tissues; however, AC011092.3 showed no significant difference. The results for AC104785.1 and AC009951.6 were contrary to those from the TCGA database analysis, which may be due to the small sample size and high individual variability.

## 4 Discussion

In the field of cancer treatment, new discoveries continuously bring hope to patients. Recently, a groundbreaking study revealed a novel form of cell death called “disulfide death,” which may provide a new strategy for cancer therapy. This type of cell death differs from common apoptosis and ferroptosis, characterized by abnormal accumulation of disulfide bonds between actin cytoskeletal proteins in cancer cells, leading to the collapse of the cell skeleton and eventual cell death. This process is particularly prominent in cancer cells with high expression of SLC7A11, especially under conditions of glucose deprivation (10). The discovery of disulfide death not only enriches our understanding of cell death mechanisms but also offers new potential targets for cancer treatment. Studies indicate that the features of disulfide death can be used to predict the prognosis of various types of tumors, including BLCA (45) and lung adenocarcinoma (46). LncRNAs play an important role in regulating the malignant behavior of tumor cells and have been proven to be potential biomarkers and targets for cancer diagnosis and therapy (36). However, DRLs are largely unknown. In this study, we identified six DRLs and established a prognostic risk model for patients with BLCA.

In the field of cancer research, a deep understanding of the tumor microenvironment and immune regulation mechanisms is crucial for developing effective treatment strategies. Our study leveraged information from public databases such as TCGA and GEO, and through the analysis of single-cell sequencing data, we identified 12 DEGs in BLCA. This research not only provided new insights into gene expression in BLCA but also revealed the critical roles of MIF-(CD74+CD44) and MIF-(CD74+CXCR4) signals in regulating communication-induced DEG expression through intercellular communication analysis. The study found that the MIF-(CD74+CD44) complex play a significant role in tumor development by promoting immune cell migration and activation (47). Moreover, MIF, through binding with CD74 and CXCR4, facilitates tumor cell migration and invasion and regulates the immunosuppressive state in the tumor microenvironment (41). These findings offer a fresh perspective on understanding the complexity of the tumor microenvironment and may hold significant implications for identifying new therapeutic targets.

Subsequently, 144 co-expressed lncRNAs were identified through the 12 DEGs. Then, prognostic lncRNAs were obtained through UniCox analysis, and a risk model composed of 6 lncRNAs (C1RL-AS1, GK-AS1, AC134349.1, AC104785.1, AC011092.3, and AC009951.6) was constructed through LASSO regression and multivariate regression analysis. The reliability of this model was confirmed by validating two clinical characteristic indicators of risk sets. By combining relevant risk factors, we generated a predictive nomogram for clinical application. Our predictive model effectively distinguished high-risk and low-risk patients in the entire cohort and subgroups, with higher risk samples having poorer OS and PFS. Tumor staging reflects the progression and severity of the disease, and our model is as sensitive as tumor staging in predicting 1-year, 3-year, and 5-year survival rates. The enrichment analysis of DEGs revealed the activation of many signaling pathways associated with tumorigenesis, such as cell adhesion molecules cams, focal adhesion, regulation of actin cytoskeleton, and systemic lupus erythematosus. Therefore, this model associated with DRLs is an accurate and reliable prognostic predictor for BLCA patients.

The tumor microenvironment (TME) is a complex ecosystem that encompasses not only tumor cells but also immune cells, stromal cells, cytokines, and other molecular components. This dynamic system is closely associated with the initiation, growth, metastasis of tumors, and their response to treatment (48, 49). In BLCA, the role of the TME is particularly crucial. It not only influences tumor recurrence and progression but also has the potential to serve as a therapeutic target (45). In our study, we found that most immune cells were enriched in the high-risk group, including Monocytes, Macrophages M0, Macrophages M2, and Mast cells resting, with significant differences between the high-risk and low-risk groups (p < 0.01). In the low-risk group, T cells regulatory (Tregs) and Dendritic cells activated were positively correlated. Monocytes and Macrophages M0/M2 are key cells in inflammatory responses, and their accumulation in tissues may be associated with inflammatory diseases or an immunosuppressive state in the tumor microenvironment (50). M2 macrophages, in particular, are associated with an anti-inflammatory and tumor growth-promoting environment. The high content of Tregs in the low-risk group may be related to a stronger immunosuppressive environment; Tregs help maintain immune tolerance and prevent excessive immune responses, but in the tumor microenvironment, they may also aid the tumor in evading immune surveillance (51). These findings collectively indicate that there is a strong correlation between DRLs and immune responses.

Immune checkpoint inhibitors (ICI) have revolutionized cancer treatment by demonstrating significant therapeutic effects across various types of tumors, making them a first-line adjuvant treatment choice for many cancer types. The TIDE (Tumor Immune Dysfunction and Exclusion) method is an advanced tool used to predict BLCA patients’ response to ICB and the likelihood of immune escape. Research indicates that high-risk patients may be more prone to immune escape, which is associated with their lower tumor mutational burden (TMB) (**Figure 10A**) (52, 53). TMB is a crucial indicator for predicting favorable responses to immunotherapy because it is often linked to the generation of more neoantigens by tumor cells, which can be recognized by the immune system, eliciting an effective immune response (54). Moreover, high-risk patients have shown better responses to certain drugs such as cisplatin, Ribociclib, SB216763, Obatoclax Mesylate, Venetoclax, among others. This suggests that despite facing higher risks of immune escape, these patients may exhibit enhanced responses to specific medications, possibly linked to their immune status and tumor biology. By integrating this information, more precise prognostic assessments and personalized treatment recommendations can be offered to BLCA patients, thereby increasing treatment success rates and improving patients’ quality of life.

Overall, the prognostic model we constructed with DRLs can independently assess the clinical outcomes of BLCA patients and is closely associated with their survival and response to immunotherapy and chemotherapy drugs. Our research aim is to provide a scientific basis for the molecular mechanisms of DRLs in BLCA and their prospects for application in clinical treatment. However, this study also faces some challenges; in addition to the limitations of sample size, the model lacks external validation, The study did not include confounding factors such as medical treatment history and lifestyle, and future research will need to integrate multi-omics data (clinical follow-up, metabolomics) to refine the model. To further understand the underlying mechanisms of DRLs, for instance, AC134349.1 may influence cysteine metabolism by regulating the SLC7A11-related pathway. Future studies will employ techniques such as CRISPR screening and RNA pull-down to verify its interaction with DRGs and explore its regulatory role in chemoresistance or immunotherapy. Additionally, clinical translation efforts include the development of liquid biopsy biomarkers or targeted therapies based on DRLs to enhance personalized treatment of BLCA.

## 5 Conclusion

In conclusion, by analyzing the DRGs between cells, we identified DEGs and based on these, constructed a prognostic model for BLCA patients based on DRLs. This model can effectively predict the survival outcomes of BLCA patients and distinguish between high-risk and low-risk groups, emphasizing their association with immune responses and chemotherapy sensitivity.

## Data Availability

The datasets presented in this study can be found in online repositories. The names of the repository/repositories and accession number(s) can be found in the article/supplementary material.
